# Validation study of randomly selected cases of PTSD diagnoses identified in a Swedish regional database compared with medical records: is the validity sufficient for epidemiological research?

**DOI:** 10.1136/bmjopen-2019-031964

**Published:** 2019-12-23

**Authors:** Anna-Clara Hollander, Klara Askegård, Claudia Iddon-Escalante, Emily A Holmes, Susanne Wicks, Christina Dalman

**Affiliations:** 1 Department of Global Public Health Sciences, Karolinska Institutet, Stockholm, Sweden; 2 Psykiatri Södra, Administration, Stockholm, Sweden; 3 Department of Clinical Neuroscience, Karolinska Institutet, Stockholm, Sweden; 4 Department of Psychology, Uppsala University, Sweden, Uppsala, Sweden; 5 Centre for Epidemiology and Community Medicine, Region Stockholm, Stockholm, Sweden

**Keywords:** epidemiology, psychiatry, public health

## Abstract

**Objectives:**

In Sweden, the patients’ diagnoses are recorded in administrative registers. The research value of these registers is determined by their diagnostic validity, that is, if the diagnosis recorded meets the relevant diagnostic criteria. The aim of the study was to assess the validity of post-traumatic stress disorder (PTSD)-diagnoses as compared with case notes in medical records (MRs) and to test if there was a difference in validity by gender, migration status and those with and without psychotic symptoms. We hypothesised that the validity would be sufficient, using both *Diagnostic and Statistical Manual of Mental Disorders* (DSM)-IV and DSM-5 but higher according to DSM-IV than DSM-5, and that the validity would be the same for men and women, but different for Swedish-born and migrants, and for those with and without psychotic symptoms.

**Design and setting:**

A validation of the register-diagnoses using MRs from treatment centres within the Region of Stockholm to examine whether patients with a register-diagnosis of PTSD fulfilled DSM criteria of PTSD according to the case notes in their MRs.

**Participants:**

A random sample of 187 patients aged 18–64, who had been diagnosed with PTSD (F43.1 in the ICD-10) were drawn from the Region of Stockholm’s MR database 2013–2015.

**Primary outcome measure:**

Validity of the PTSD diagnoses according to DSM-IV and DSM-5 as proportions of true positives with 95% CI.

**Results:**

The hypothesised sufficient validity of the PTSD diagnoses was confirmed. Although the point-estimates for DSM-IV were higher than for DSM-5, the hypothesis that there would be significant differences in validity between DSM-IV and DSM-5 was not confirmed. There were no significant validity differences by gender, migration status and for those with and without psychotic symptoms.

**Conclusions:**

This study has found that validity of the PTSD diagnoses in the register of the Region of Stockholm to be sufficient for epidemiological research.

**Strengths and limitations of this study**

- Randomly selected sample of cases with a post-traumatic stress disorder (PTSD) diagnosis from the comprehensive register of the Stockholm region with an almost complete coverage and mixed urban populations.
- Thorough review of the medical records (MRs) performed by two medical doctors.
- The two medical doctors reviewing MRs had a high coherence.
- The method of validating diagnosis by reviewing MRs carries a risk of misinterpretation when reading another medical doctor’s case notes.
- The Stockholm population is an urban population, hence, there might be specific aspects of PTSD in rural areas that are not taken into account.

## Introduction

Post-traumatic stress disorder (PTSD) can develop after a person has been exposed to exceptionally threatening or horrifying events which qualify as ‘psychological trauma’.^1^ Symptoms of PTSD, continuing more than a month after the event, include involuntary and intrusive upsetting memories of the traumatic event, thoughts, feelings, or dreams related to the events, mental or physical distress to trauma-related cues, attempts to avoid trauma-related cues, alterations in how a person thinks and feels, and increased arousal.^2 3^ There is a strong case for the cross-cultural validity of PTSD,^4^ still, estimates of PTSD prevalence differ greatly between countries.^4^ In a study of PTSD in European countries, the highest prevalence of PTSD was found in war-torn Croatia, followed by the Netherlands, the UK, France and Germany.^5^ The samples included in this comparison were small and survey-based with varying attrition.

Psychotic disorders and PTSD are different in many ways but arguably have some similarities in terms of specific symptoms.^6^ Some avoidance behaviours in PTSD resemble safety-seeking behaviours or negative symptoms in psychosis.^6^ Hallucinations in psychosis have similarities to the experience of flashbacks and intrusive images and bodily sensations in PTSD.^6^ There is an ongoing discussion about the relationship between PTSD and psychosis.^7^ Studies of refugees have, so far, often had a focus on PTSD, however, a study from 2016 showed that in particular male refugees also have an increased risk of non-affective psychosis.^8^ This study has intensified an already ongoing debate regarding the validity of PTSD diagnoses among male and female migrants, especially with psychosis, as for mental health professionals, different cultural variation in presentation of psychiatric symptoms contributes to risk of being misdiagnosed.^9^

In Scandinavia and Finland, visits to psychiatric care are recorded in local and national administrative registers covering the entire population (also known as population-based registers). Such registers have benefited mental health research immensely.^10^ Central understanding regarding major mental disorders, for instance, schizophrenia, bipolar disorder, suicide, autism spectrum disorders (ASD) would not exist without the use of population based registers.^10^ When using these administrative registers created for generic purposes in research it is crucial to assess the quality of the information they contain, that is, testing the diagnostic validity.^11^ Diagnostic validity refers, in this context, to the accuracy of a diagnose, if the diagnoses recorded meets the relevant diagnostic criteria, measured as proportions of true positives. The diagnose is considered valid if it corresponds to the diagnostic criteria according to either *International Classification of Diseases* (ICD)^12^ or *Diagnostic and Statistical Manual of Mental Disorders* (DSM).^2 3 12^ In Sweden diagnoses are registered according to the ICD-10. The share of cases with a valid diagnosis differ for different diagnoses and for different registers but in Sweden the population registers who have been validated have had a validity around 85%–95%,^11^ hence, this level can be considered as a sufficient validity. Validation studies have been performed for a range of specific psychiatric diagnoses, for example, schizophrenia, ASD, bipolar disorder and so on,^11^ in the Swedish registers, but hitherto the diagnosis of PTSD has not been validated. A small validation study of PTSD was performed in Denmark 2015 testing the validity of PTSD^13^ defined according to ICD-10.^12^ The study used 18 cases of PTSD from the Danish Psychiatric Central Research Register and found a positive predictive value (ppv) of 83%. The PTSD diagnosis are of special interest since the DSM criteria for PTSD have been changed recently. Valid PTSD-diagnoses that can be used in population-based register studies could improve understanding of the prevalence and incidence of PTSD.

The first aim of the current study is to assess the validity of PTSD-diagnoses in a regional register as compared with case notes in medical records (MRs) according to the DSM-IV and DSM-5 criterion, in order to determine if the register diagnoses are of sufficient quality for epidemiological research. We hypothesise that the validity of PTSD-diagnoses will be sufficient, using both DSM-IV and DSM-5 but that the validity would be higher according to DSM-IV than the DSM-5, as for the time frame chosen more clinicians would have been familiar with DSM-IV. The second aim is to test if there is a difference in the validity by gender, migration status and among the patients with and without psychotic symptoms. We hypothesise that the validity will be the same for men and women, but different for Swedish-born and migrants and for those with and without psychotic symptoms, with a higher validity for Swedish born and those without psychotic symptoms.

## Method

### Setting and design

The design of the validation was to examine whether patients with register-diagnoses of PTSD fulfilled the DSM criteria of PTSD according to the case notes in their MRs. We chose to assess the diagnoses in accordance with the DSM-system since it has become the global standard in psychiatric research and contains specific criteria for each diagnosis. MRs were retrieved from four local specialist psychiatric treatment centres within the Region of Stockholm in Sweden: Psykiatri Södra, Psykiatri Sydväst, Norra Stockholms Psykiatri and Psykiatri Nordväst.

### Population and data source

We selected a random sample of 200 patients from the approximately 2000 eligible patients in the register, aged 18–64, who had been diagnosed with PTSD (F43.1 according to ICD-10^12^) at one of the above-mentioned local centres as a primary or secondary diagnosis according to the Region of Stockholm’s healthcare register 2013–2015. The reason for including 200 patients were to have the statistical power to do the subgroup calculations even if the validity was found to be low. The MRs of these patients were retrieved from the Region of Stockholm’s electronic MR database *Take Care,* that was introduced within the Region of Stockholm in 2008 and used by the majority of clinics by year 2010. Thirteen MRs were not possible to retrieve due to, for instance, hidden identity of the MR holder. These MRs were deducted from the total number of MRs. The size of the MRs ranged from 1 to 100 pages, sometimes from different psychiatric caregivers.

### Validation procedure

Two medical doctors, in their psychiatric specialty training, revised 90 MRs each and 20 together to cross-validate their judgements (total n=200). They reviewed both symptoms for PTSD, and psychosis. Considering that DSM-IV was used until late 2014, when DSM-5 was published in Swedish, the PTSD diagnostic criteria was scrutinised for both DSM-IV and DSM-5. Twenty patients were cross validated by both the clinicians in order to calculate the degree of coherence between the two doctors, and in conjunction with supervision from a specialist in PTSD. When the MRs included results from a Mini International Neuropsychiatric Interview (M.I.N.I.)^14^ concluding that the patient fulfilled the criteria for a PTSD diagnosis, this was considered as the criteria were fulfilled, although each criterion was not commented on specifically. The clinicians also reviewed the MRs for positive or negative symptoms of psychosis, whether the patient fulfilled a psychosis diagnosis according to M.I.N.I. or whether the patient had received any diagnosis of psychosis in the MR (henceforth, referred to as psychosis according to MR).

### Subgroups

The diagnoses were compared by gender according to the MR, migration status (born in Sweden or not) according to the MR and by psychosis according to notes in the MR. The patient was classified as having psychosis according to notes in the MR by the reviewer if the patients either fulfilled the criteria of both hallucinations and delusions or were diagnosed with a psychotic disorder according to M.I.N.I.^14^ or if the patient was classified as having a suspected psychotic diagnosis.

### Statistical methods

We calculated the degree of coherence between raters, in per cent and validity as ppv among the register diagnoses with 95% CI, ppv is defined as number of patients with a PTSD register diagnosis confirmed in the MRs divided by the total number of patients with a PTSD register diagnosis that we were able to validate against MRs. The differences between subgroups were compared using χ^2^ tests.

In order for a DSM-criteria to be counted as fulfilled, the criteria needed to be explicitly mentioned in the MR. However, criteria C for PTSD (the avoidance criteria) seemed sometimes not to be mentioned unless it was not fulfilled (ie, ‘the patient do not seem to avoid related cues’). An additional test of validity was made, were we counted criteria C to be fulfilled each time it was not specifically described as not to be fulfilled.

## Results

### Study population

Due to either protected identities or inaccessible records, MRs could not be validated for 13 persons out of the 200 selected. Since we could not assess the validity of PTSD among these patients, they were excluded from the study. Altogether 187 register diagnoses were validated in the final sample, which included more women than men and more Swedish born than migrants (see table 1). The coherence between the two medical doctors validating the MRs was 80% (95% CI 62% to 98%) for DSM-IV and 85% (95% CI 69% to 100%) for DSM-5.

**Table 1:** Demographic description of the retrieved cases of PTSD from the register

		N (%)
**Total number of selected cases**		200 (100)
Number of accessible records that is, final sample		187 (94)
**Demographics of the final sample**		
Gender according to MR	Men	68 (36)
	Women	119 (64)
Migration status according to MR	Swedish born	108 (58)
	Foreign born	79 (42)
Psychosis according to notes in the MR	Yes	16 (9)
	No	171 (91)

MRs, medical records; PTSD, post-traumatic stress disorder.

### Validity of PTSD according to DSM-IV and DSM 5

Out of 187 patients, 84% (95% CI 79% to 90%) fulfilled the criteria of PTSD according to DSM-IV and 75% (95% CI 69% to 82%) of the patients qualified for a PTSD diagnosis according to DSM-5 (see table 2). There were 29 (for DSM-IV) and 46 (for DSM-5) false positive cases. Among the false positive cases, there were no transferring errors (ie, complete mistake with unrelated diagnoses miscoded as PTSD) as all cases had some symptoms relating to PTSD, but still did not fulfil the PTSD criteria.

**Table 2:** Validity, positive predictive value (ppv), of the PTSD-diagnoses in the register among the 187 accessible MRs and the additional test of validity counting cases with inadequate information on criteria C as true positives

According to the 187 MRs	DSM-IV		DSM-5	
	N	ppv (95% CI)	N	ppv (95% CI)
True positive cases	158	84 (79 to 90)	141	75 (69 to 82)
Additional test of validity				
True positive cases	168	90 (86 to 94)	154	82 (77 to 88)

DSM, *Diagnostic and Statistical Manual of Mental Disorders*; MRs, medical records; PTSD, post-traumatic stress disorder.

The false positive cases were primarily due to two reasons. Either not fulfilling the central criteria of being exposed to a trauma specific event (criteria A) or not describing any signs of avoidance, criteria C. According to the DSM-IV criteria A was fulfilled 93% of the times and 92% the DSM-5. Regarding criteria C, 88% fulfilled the DSM-IV, and 84% the DSM-5 fulfilled criteria C. For more information on the per cent of the cases fulfilling each specific PTSD criteria in detail, see online supplementary appendix A. In the additional test of validity test criteria C was calculated as fulfilled each time it was not specifically described as not to be fulfilled. When counting validity according to the additional test of validity test the validity of the PTSD-diagnosis was 90% (95% CI 86% to 94%) for DSM-IV and, 82% (95% CI 77% to 88%) for DSM-5. There were no significant differences in terms of validity by gender, migration status and psychosis according to notes in the MR (see table 3).

10.1136/bmjopen-2019-031964.supp1 Supplementary data

**Table 3:** Validity, positive predictive value (ppv), of the PTSD-diagnoses in the register according to accessible MRs by gender, migration status and psychosis according to notes in MR for DSM-IV and DSM-5 and p value for the differences using χ^2^ or Fishers exact test

	DSM-IV		DSM-5	
	ppv (95% CI)	P value	ppv (95% CI)	P value
Gender according to MR				
Men	87 (79 to 95)	χ^2^ test	78 (68 to 88)	χ^2^ test
Women	83 (76 to 90)	0.5163	74 (66 to 82)	0.5421
Migration status according to MR				
Swedish born	82 (74 to 91)	χ^2^ test	72 (62 to 82)	χ^2^ test
Foreign born	86 (79 to 93)	0.4745	78 (70 to 86)	0.3776
Psychosis according to notes in MR				
Yes	94 (82 to 100)	Fisher’s exact test	88 (71 to 100)	Fisher’s exact test
No	84 (78 to 89)	0.4737	74 (68 to 81)	0.3648

DSM, *Diagnostic and Statistical Manual of Mental Disorders*; MRs, medical records; PTSD, post-traumatic stress disorder.

## Discussion

In this study, the first to assess the validity of PTSD-diagnoses in a Swedish population-based register, the hypothesised sufficient validity of the PTSD-diagnoses was confirmed because the ppv of the diagnoses were between 75% and 90%. Although the point-estimates for DSM-IV was higher than for DSM-5 the hypothesis that it would be a significant difference in validity between DSM-IV and DSM-5 was not confirmed. There were neither any significant validity differences between men and women, Swedish born and migrants, nor for those with and without psychosis according to notes in the MR.

A strength of the study is that the sample of persons with a PTSD diagnosis was randomly selected from the comprehensive register of the Stockholm country council representing four major psychiatric treatment centres with an almost complete coverage and mixed urban populations, and this limits sampling bias. Another strength is that the clinicians reviewing MRs had a high coherence. A limiting factor with the method of validating diagnosis by reviewing MRs is a risk of misinterpretation when reading the case notes of other medical professionals. The ultimate gold standard of validation is always clinical interviews; however, using MRs is a standard method that as the data are more easily available, more cost effective and less intrusive for patients.

Criteria C for DSM-IV (‘Persistent avoidance of stimuli associated with the trauma and numbing of general responsiveness’), and criteria D for DSM-5 (‘Negative thoughts or feelings that began or worsened after the trauma’) were considered to be non-stringent by the medical doctors reading the MRs as there is a risk of mistaking these symptoms for a symptom of a depressive state or vice-versa. The Stockholm population is an urban population, hence, there might be specific rural aspects of PTSD that are not taken into account. It is also important to note that these are clinical cases; hence, using PTSD in the register only accounts for clinical cases of PTSD and will by definition miss patients who do have PTSD but does not present in the healthcare system. Another weakness was that the migrant status was defined according to birthplace classified according to the MRs, and not according to a population data base were all places of births are recorded. Most MRs are very detailed in terms of background information however there might be instances were a migration background is not mentioned.

Just like the smaller Danish validation study^13^ from 2015 testing the validity of PTSD defined according ICD-10^12^ we found a sufficient validity of the PTSD diagnoses. The accuracy we found for DSM-IV (84%) was similar to that of the Danish study (83%) but a little bit lower for DSM-5 (75%), which is perhaps not surprising as it had only just been started to be used in the time frame of our investigation. However, a validation study from the USA including 4777 veterans comparing Department of Veterans Affairs administrative data with the self-assessment questioner PTSD Checklist had the same validity as our results as when diagnosed using DSM-5 (75%).^15^ In DSM-5 the American Psychiatric Association updated the diagnostic criteria for PTSD. The key changes between DSM-IV and DSM-5 are that the trauma criterion is different and that feelings such as intense fear, hopelessness, or horror, are removed from DSM-5.^16^ Another change is that one criterion was made into two; one avoidance criteria and one criterion for negative alterations in cognitions and mood. This put more emphasis on avoidance symptoms in the DSM-5 version. Two criterion have also been added, one regarding negative thoughts or feelings and one regarding trauma-related arousal and reactivity, requiring that they began or worsened after the trauma. The validity differences in our study between DSM-IV and DSM-5 were small and not statistically significant and are possibly associated with the altered criteria in DSM-5. A study in the US shows that the changes in the diagnostic criteria for PTSD in the DSM from IV to 5 have had a minimal impact on prevalence.^16^ Still, the national PTSD prevalence estimates in the USA have been slightly lower (ca 1%), for both lifetime, and past 12-month PTSD when using DSM-5 as compared with DSM-IV.^16^ In the same study, the differences between DSM-IV and DSM-5 seem to be due to the exclusion of the ‘sudden unexpected death of a loved one’ as criteria for trauma in the DSM-5 and that DSM-5 is more explicit regarding the avoidance criteria.^16^ The altered trauma criteria and the more explicit avoidance criteria seems to make the greatest difference between DSM-IV and DSM-5 in our study too.

This study has found that the validity of the PTSD diagnoses in the healthcare registers of the Region of Stockholm is sufficient for epidemiological research. The register can be used for population-based register studies of PTSD for men and women, Swedish born and migrants and person with and without psychosis according to notes in the MR and studies generated from its use have the potential to greatly improve understanding of the prevalence and incidence of PTSD and risk factors of the diagnosis.

## Supplementary Material

Reviewer comments

Author's manuscript
