# Supplementary material for: Validation study of randomly selected cases of PTSD diagnoses identified in a Swedish regional database compared with medical records: is the validity sufficient for epidemiological research?
Source: BMJ Open. 2019 Dec 23;9(12):e031964. doi: 10.1136/bmjopen-2019-031964 (PMC7008445; doi:10.1136/bmjopen-2019-031964)
Supplement: Supplementary data [file bmjopen-2019-031964supp001.pdf]

Appendix A: Each specific diagnose criteria of PTSD according to the DSM-IV and DSM-5 and the percent fulfilment of each of these criteria according to the MRs of the 187 MRs

	**DSM-IV**		**DSM-5**	
	Criteria		Criteria	
A	Exposed to a traumatic event	93%	The person was exposed to: death, threatened death, actual or threatened serious injury, or actual or threatened sexual violence	92%
B	The traumatic event is persistently re-experienced	96%	The traumatic event is persistently re-experienced	96%
C	Persistent avoidance of stimuli associated with the trauma and numbing of general responsiveness	88%	Avoidance of trauma-related stimuli after the trauma	84%
D	Persistent symptoms of increased arousal	96%	Negative thoughts or feelings that began or worsened after the trauma	94%
E	Symptoms last for more than 1 month.	98%	Trauma-related arousal and reactivity that began or worsened after the trauma	97%
F	The disturbance causes clinically significant distress or impairment	98%	Symptoms last for more than 1 month.	98%
G		n.a.	Symptoms create distress or functional impairment	97%
H		n.a.	Symptoms are not due to medication, substance use, or other illness.	93%
