# Supplementary material for: Validation study of randomly selected cases of PTSD diagnoses identified in a Swedish regional database compared with medical records: is the validity sufficient for epidemiological research?
Source: BMJ Open. 2019 Dec 23;9(12):e031964. doi: 10.1136/bmjopen-2019-031964 (PMC7008445; doi:10.1136/bmjopen-2019-031964)
Supplement: Author's manuscript [file bmjopen-2019-031964.draft_revisions.pdf]

BMJ Open is committed to open peer review. As part of this commitment we make the peer review history of every article we publish publicly available.

When an article is published we post the peer reviewers' comments and the authors' responses online. We also post the versions of the paper that were used during peer review. These are the versions that the peer review comments apply to.

The versions of the paper that follow are the versions that were submitted during the peer review process. They are not the versions of record or the final published versions. They should not be cited or distributed as the published version of this manuscript.

BMJ Open is an open access journal and the full, final, typeset and author-corrected version of record of the manuscript is available on our site with no access controls, subscription charges or pay-per-view fees (http://bmjopen.bmj.com).

If you have any questions on BMJ Open's open peer review process please email info.bmjopen@bmj.com

BMJ Open

A validation study of cases of PTSD diagnoses identified in a Swedish regional database - Is the validity sufficient for epidemiological research?

Journal:	*BMJ Open*
Manuscript ID	bmjopen-2019-031964
Article Type:	Research
Date Submitted by the Author:	28-May-2019
Complete List of Authors:	Hollander, Anna-Clara; Karolinska Institutet, Department of Public Health Sciences<br>Askegård, Klara; Psykiatri Södra, Administration, Tideliusgatan 12, plan 6, 118 69 Stockholm<br>Escalante-Bernal, Claudia; Psykiatri Södra, Administration, Tideliusgatan 12, plan 6, 118 69 Stockholm<br>Holmes, Emily; Karolinska Institutet Department of Clinical Neuroscience; Uppsala University, Sweden, Department of Psychology<br>Wicks, Susanne; Karolinska Institutet, Division of Public Health Epidemiology, Department of Public Health Sciences; Centre for Epidemiology and Community Medicine, Stockholm County Council, SE-171 77 Stockholm, Sweden<br>Dalman, Christina; Karolinska Institute, Public Health Sciences; Centre for Epidemiology and Community Medicine, Stockholm County Council, SE-171 77 Stockholm, Sweden
Keywords:	EPIDEMIOLOGY, PSYCHIATRY, PUBLIC HEALTH

SCHOLARONE™
Manuscripts

Title page

Title: A validation study of cases of PTSD diagnoses identified in a Swedish regional database - Is the validity sufficient for epidemiological research?

Corresponding author: Anna-Clara Hollander, Department of Public Health Sciences, Karolinska Institutet, 171 77 Stockholm, Sweden, anna-clara.hollander@ki.se

Authors and affiliations:

Anna-Clara Hollander[1], Klara Askegård[2], Claudia Escalante[2], Emily A. Holmes[3], Susanne Wicks[1,4], Christina Dalman[1,4]

[1]Epidemiology of Psychiatric Conditions, Substance use and Social environment (EPiCSS), Department of Public Health Sciences, Karolinska Institutet, 171 77 Stockholm

[2]Psykiatri Södra, Administration, Tideliusgatan 12, plan 6, 118 69 Stockholm

[3]Emotional Mental Imagery Lab (EMIL), Department of Clinical Neuroscience, Karolinska Institutet, 171 77 Stockholm and Department of Psychology, Uppsala University, Sweden

[4]Centre for Epidemiology and Community Medicine, Stockholm County Council, SE-171 77 Stockholm, Sweden

Word count: 2536

For peer review only

Abstract

Objectives**:** In Sweden, the diagnoses of patients are recorded in administrative registers. The research value of these registers is determined by their diagnostic validity, i.e. if the diagnoses recorded meets the relevant diagnostic criteria. The aim of the study was to assess the validity of PTSD-diagnoses as compared with case notes in medical records (MR) and to test if there was a difference in validity by gender, migration status and those with and without psychotic symptoms. We hypothesize that the validity would be feasible, using both DSM-IV and DSM-5 but higher according to DSM-IV than the DSM-5, and that the validity would be the same for men and women, but different for Swedish-born and migrants, and for those with and without psychotic symptoms.

Design and setting**:** A validation of the register-diagnoses using MRs from treatment centres within the Region of Stockholm to examine whether patients with a register-diagnosis of PTSD fulfilled DSM criteria of PTSD according to the case notes in their MRs.

Participants: A random sample of 187 patients aged 18-64, who had been diagnosed with PTSD (F43.1 in the ICD-10) were drawn from the Region of Stockholm's MR database 2013 – 2015.

Primary outcome measure**:** Validity of the PTSD diagnose according to DSM-IV and DSM-5 as proportions of true positives with 95% confidence interval.

Results: The hypothesised feasible validity of the PTSD diagnose was confirmed. Although the point-estimates for DSM-IV were higher than for DSM-5, the hypothesis that it would be significant differences in validity between DSM-IV and DSM-5 was not confirmed. There were no significant validity differences by gender, migration status and for those with and without psychotic symptoms.

Conclusions: This study has found that the validity of the PTSD diagnoses in the register of the Region of Stockholm to be sufficient for epidemiological research.

Keywords: post-traumatic stress disorder, diagnostic validity, administrative registers, DSM IV, DSM-5

Strengths and limitations of this study

- Randomly selected sample of cases with a PTSD diagnosis from the comprehensive register of the Stockholm region with an almost complete coverage and mixed urban populations.
- Thorough review of the medical records performed by two medical doctors.
- The two medical doctors reviewing medical records had a high coherence.
- The method of validating diagnosis by reviewing MRs carries a risk of misinterpretation when reading another medical doctor's case notes.
- The Stockholm population is an urban population, hence there might be specific aspects of PTSD in rural areas that are not taken into account.

For peer review only - http://bmjopen.bmj.com/site/about/guidelines.xhtml

A validation study of cases of PTSD diagnoses identified in a Swedish regional database - Is the validity sufficient for epidemiological research?

Introduction

Posttraumatic stress disorder (PTSD) can develop after a person has been exposed to exceptionally threatening or horrifying events which qualify as "psychological trauma"[1]. Symptoms of PTSD, continuing more than a month after the event, include involuntary, and intrusive upsetting memories of the traumatic event, thoughts, feelings, or dreams related to the events, mental or physical distress to trauma-related cues, attempts to avoid trauma-related cues, alterations in how a person thinks and feels, and increased arousal[2 3]. There is a strong case for the cross-cultural validity of PTSD[4], still, estimates of PTSD prevalence differ greatly between countries[4]. In a study of PTSD in European countries, the highest prevalence of PTSD was found in war-torn Croatia, followed by the Netherlands, the UK, France and Germany[5]. The samples included in this comparison were small and survey-based with varying attrition.

Psychotic disorders and PTSD are different in many ways but arguably have some similarities in terms of specific symptoms[6]. Some avoidance behaviours in PTSD resemble safety-seeking behaviours or negative symptoms in psychosis[6]. Hallucinations in psychosis have similarities to the experience of flashbacks and intrusive images and bodily sensations in PTSD[6]. There is an ongoing discussion about the relationship between PTSD and psychosis[7]. Studies of refugees have, so far, often had a focus on PTSD, however, a study from 2016 showed that refugees also have an increased risk of non-affective psychosis[8]. This has intensified an already ongoing debate regarding the validity diagnoses among migrants, as for mental health professionals, different cultural variation in presentation of psychiatric symptoms contributes to risk of being misdiagnosed[9].

In Scandinavia and Finland, visits to psychiatric care are recorded in local and national administrative registers covering the entire population (also known as population-based registers). Such registers have benefited mental health research immensely[10]. Central understanding regarding major mental disorders, for instance, schizophrenia, bipolar disorder, suicide, autism spectrum disorders (ASD) would not exist without the use of population based registers[10]. When using these administrative registers created for generic purposes in research it is crucial to assess the quality of the information they contain, i.e. testing the diagnostic validity[11]. Diagnostic validity refers, in this context, to the accuracy of a diagnose, if the diagnoses recorded meets the relevant diagnostic criteria, measured as proportions of true positives. The diagnose is considered valid if it corresponds to the diagnostic criteria according to either *International Classification of Diseases* (ICD)[12] or *Diagnostic and Statistical Manual of Mental Disorders* (DSM)[2 3 12]. In Sweden diagnoses are registered according to the ICD-10. The share of cases with a valid diagnosis differ for different diagnoses and for different registers but in Sweden the population registers who have been validated have had a validity around 85-95%[11], hence this level can be considered as a feasible validity. Validation studies have been performed for a range of specific psychiatric diagnoses e.g. schizophrenia, ASD, bipolar disorder etc.[11], in the Swedish registers, but hitherto the diagnosis of PTSD has not been validated. A small validation study of PTSD was performed in Denmark 2015 testing the validity of PTSD[13] defined according to the tenth addition of ICD-10[12]. The study used 18 cases of PTSD from the Danish Psychiatric Central Research Register and found a positive predictive value of 83%. The PTSD diagnosis are of special interest since the DSM criteria for PTSD have been changed recently. Valid PTSD-diagnoses that can be used in

For peer review only - http://bmjopen.bmj.com/site/about/guidelines.xhtml

population-based register studies could improve understanding of the prevalence and incidence of PTSD.

The first aim of the current study is to assess the validity of PTSD-diagnoses in a regional register as compared with case notes in medical records (MR) according to the DSM-IV and DSM-5 criterion, in order to determine if the register diagnoses are of sufficient quality for epidemiological research. We hypothesize that the validity of PTSD-diagnoses will be feasible, using both DSM-IV and DSM-5 but that the validity would be higher according to DSM-IV than the DSM-5, as for the time frame chosen more clinicians would have been familiar with DSM-IV. The second aim is to test if there is a difference in the validity by gender, migration status and among the patients with and without psychotic symptoms. We hypothesize that the validity will be the same for men and women, but different for Swedish-born and migrants and for those with and without psychotic symptoms, with a higher validity for Swedish born and those without psychotic symptoms.

Method

Setting and design:

The design of the validation was to examine whether patients with register-diagnoses of PTSD fulfilled the DSM criteria of PTSD according to the case notes in their MRs. We chose to assess the diagnoses in accordance with the DSM-system since it has become the global standard in psychiatric research and contains specific criteria for each diagnosis. MRs were retrieved from four local specialist psychiatric treatment centres within the Region of Stockholm in Sweden: Psykiatri Södra, Psykiatri Sydväst, Norra Stockholms Psykiatri och Psykiatri Nordväst.

Population and data source:

We selected a random sample of 200 patients from the approximately 2000 eligible patients in the register, aged 18-64, who had been diagnosed with PTSD (F43.1 according to ICD-10[12]) at one of the above-mentioned local centres as a primary or secondary diagnosis according to the Region of Stockholm's health care register 2013 – 2015. The MRs of these patients were retrieved from the Region of Stockholm's electronic MR database *Take Care,* that was introduced within the Region of Stockholm in 2008 and used by the majority of clinics by year 2010. Thirteen MRs were not possible to retrieve due to, for instance, hidden identity of the MR holder. These MRs were deducted from the total number of MRs. The size of the medical records ranged from 1 - 100 pages, sometimes from different psychiatric caregivers.

Validation procedure:

Two medical doctors, in their psychiatric specialty training, revised 90 MRs each and 20 together to cross-validate their judgements (total n=200). They reviewed both symptoms for PTSD, and psychosis. Considering that DSM-IV was used until late 2014, when DSM-5 was published in Swedish, the PTSD diagnostic criteria was scrutinised for both DSM IV and DSM-5. Twenty patients were cross validated by both the clinicians in order to calculate the degree of coherence between the two doctors, and in conjunction with supervision from a specialist in PTSD. When the MRs included results from a Mini International Neuropsychiatric Interview (M.I.N.I.)[14] concluding that the patient fulfilled the criteria for a PTSD diagnosis, this was considered as the criteria were fulfilled, although each criterion was not commented on specifically. The clinicians also reviewed the MRs for positive or negative symptoms of psychosis, whether the patient fulfilled a psychosis diagnosis according to

For peer review only - http://bmjopen.bmj.com/site/about/guidelines.xhtml

MINI or whether the patient had received any diagnosis of psychosis in the MR (henceforth referred to as psychosis according to MR).

Subgroups

The diagnoses were compared by gender according to the MR, migration status (born in Sweden or not) according to the MR and by psychosis according to MR. The patient was classified as having psychosis according to MR by the reviewer if the patients either fulfilled the criteria of both hallucinations and delusions or were diagnosed with a psychotic disorder according to M.I.N.I[14] or if the patient was classified as having a suspected psychotic diagnosis.

Statistical methods:

We calculated the degree of coherence between raters, in percent and validity as proportions of true positives among the register diagnoses with 95% confidence interval (95% CI). The differences between subgroups were compared using by chi-square tests.

In order for a DSM-criteria to be counted as fulfilled, the criteria needed to be explicitly mentioned in the MR. However, criteria C for PTSD (the avoidance criteria) seemed sometimes not to be mentioned unless it was not fulfilled (i.e. "the patient do not seem to avoid related cues"). An additional test of validity was made, were we counted criteria C to be fulfilled each time it was not specifically described as not to be fulfilled.

Ethics:

The study was approved by the Regional Ethical Review Board in Stockholm (Dnr 2016/1537-32).

Results:

Study population

Due to either protected identities or inaccessible records, MRs could not be validated for 13 persons out of the 200 selected. Since we could not assess the validity of PTSD among these patients, they were excluded from the study. Altogether 187 register diagnoses were validated in the final sample, which included more women than men and more Swedish born than migrants, see table 1. The coherence between the two medical doctors validating the MRs was 80% (95% CI =72-88%) for DSM-IV and 85% (95% CI=78-92%) for DSM-5.

Table 1: Demographic description of the retrieved cases of PTSD from the register

		**N (%)**
**Total number of selected cases**		200 (100%)
Number of accessible records i.e. final sample		187 (94%)
**Demographics of the final sample**		
Gender according to MR	**Men**	68 (36%)
	**Women**	119 (64%)
Migration status according to MR	**Swedish born**	108 (58%)
	**Foreign born**	79 (42%)
Psychosis according to MR	**Yes**	16 (9%)
	**No**	171 (91%)

The validity of PTSD according to DSM IV and DSM 5

For peer review only - http://bmjopen.bmj.com/site/about/guidelines.xhtml

Out of 187 patients, 84% (95% CI =77-91%) fulfilled the criteria of PTSD according to DSM-IV and 75% (95% CI =67-83%) of the patients qualified for a PTSD diagnosis according to DSM-5, see table 2. There were 29 (for DSM-IV) and 46 (for DSM-5) false positive cases. Among the false positive cases, there were no transferring errors (i.e. complete mistake with unrelated diagnoses miscoded as PTSD) as all cases had some symptoms relating to PTSD, but still did not fulfil the PTSD criteria.

The false positive cases were primarily due to two reasons. Either not fulfilling the central criteria of being exposed to a trauma specific event (criteria A) or not describing any signs of avoidance, criteria C. According to the DSM-IV criteria A was fulfilled 93% of the times and 92% the DSM-5. Regarding criteria C, 88% fulfilled the DSM-IV, and 84% the DSM-5 fulfilled criteria C. For more information on the percent of the cases fulfilling each specific PTSD criteria in detail, see Appendix A. In the additional test of validity test criteria C was calculated as fulfilled each time it was not specifically described as not to be fulfilled. When counting validity according to the additional test of validity test the validity of the PTSD-diagnosis was 88% (95% CI =81-95%) for DSM- IV and, 79% (95% CI =71-87%) for DSM- 5. There were no significant differences in terms of validity by gender, migration status and psychosis according to MR, see table 3.

<u>Table 2:</u> Validity of the PTSD-diagnoses in the register among the 187 accessible MRs and the additional test of validity counting cases with inadequate information on criteria C as true positives

	**DSM-IV**		**DSM-5**	
**According to the 187 MRs**	n	% (95% CI)	n	% (95% CI)
True positive cases	158	84 (77-91)	157	75 (67-83)
Additional test of validity				
True positive cases	166	88 (81-95)	148	79 (71-87)

<u>Table 3:</u> Validity of the PTSD-diagnoses in the register according to accessible MRs by gender, migration status and psychosis according to MR for DSM IV and DSM-5 and p-value for the differences using Chi-square or Fishers exact test.

		**DSM-IV**		**DSM-5**	
		**valid**	**P-value**	**valid**	**P-value**
		% (95% CI)		% (95% CI)	
**Gender according to MR**	Men	87 (80-94)	Chi-square Test	78 (70-86)	Chi-square Test
	Women	83 (76-90)	0.5163	74 (66-82)	0.5421
**Migration status according to MR**	Swedish born	82 (74-90)	Chi-square Test	72 (64-80)	Chi-square Test
	Foreign born	86 (79-93)	0.4996	78 (70-86)	0.3298
**Psychosis according to MR**	Yes	94 (87-100)	Fisher's Exact Test	88 (81-95)	Fisher's Exact Test
	No	83 (76-90)	0.1808	73 (65-81)	0.1197

Discussion

In this study, the first to assess the validity of PTSD-diagnoses in a Swedish population-based register according to both DSM-IV and DSM-5, the hypothesised feasible validity of the PTSD diagnoses was confirmed because the proportion of valid diagnoses were between 75-88%. Although the point-estimates for DSM-IV was higher than for DSM-5 the hypothesis that it would be a significant difference in validity between DSM-IV and DSM-5 was not confirmed. There were neither any

For peer review only - http://bmjopen.bmj.com/site/about/guidelines.xhtml

significant validity differences between men and women, Swedish born and migrants, nor for those with and without psychosis according to MR.

A strength of the study is that the sample of persons with a PTSD diagnosis was randomly selected from the comprehensive register of the Stockholm country council representing four major psychiatric treatment centres with an almost complete coverage and mixed urban populations, and this limits sampling bias. Another strength is that the clinicians reviewing medical records had a high coherence. A limiting factor with the method of validating diagnosis by reviewing MRs is a risk of misinterpretation when reading the case notes of another medical professionals. The ultimate gold standard of validation is always clinical interviews; however, using MRs is a standard method that as the data is more easily available, more cost effective, and less intrusive for patients.

Criteria C for DSM-IV ("Persistent avoidance of stimuli associated with the trauma and numbing of general responsiveness"), and criteria D for DSM-5 ("Negative thoughts or feelings that began or worsened after the trauma") were considered to be non-stringent by the medical doctors reading the MRs as there is a risk of mistaking these symptoms for a symptom of a depressive state or vice-versa. The Stockholm population is an urban population, hence there might be specific rural aspects of PTSD that are not taken into account. It is also important to note that these are clinical cases; hence, using PTSD in the register only accounts for clinical cases of PTSD and will by definition miss patients who do have PTSD but does not present in the health care system. Another weakness was that the migrant status was defined according to birthplace classified according to the MRs, and not according to a population data base were all places of births are recorded. Most MRs are very detailed in terms of background information however there might be instances were a migration background is not mentioned.

Just like the smaller Danish validation study[13] from 2015 testing the validity of PTSD defined according ICD-10[12] we found a feasible validity of the PTSD diagnoses. The accuracy we found for DSM-IV (85%) was similar to that of the Danish study (83%) but a little bit lower for DSM-5 (75%), which is perhaps not surprisingly as it had only just been started to be used in the time frame of our investigation. However, a validation study from the US including 4777 veterans comparing Department of Veterans Affairs administrative data with the self-assessment questioner PTSD Checklist had the same validity as our results as when diagnosed using DSM-5 (75%)[15]. In DSM-5 the American Psychiatric Association updated the diagnostic criteria for PTSD. The key changes between DSM-IV and DSM-5 are that the trauma criterion is different and that feelings such as intense fear, hopelessness, or horror, are removed from DSM-5[16]. Another change is that one criterion was made into two; one avoidance criteria, and one criterion for negative alterations in cognitions and mood. This put more emphasis on avoidance symptoms in the DSM-5 version. Two criterions have also been added, one regarding negative thoughts or feelings and one regarding trauma-related arousal and reactivity, requiring that they began or worsened after the trauma. The validity differences in our study between DSM-IV and DSM-5 were small and not statistically significant and are possibly associated with the altered criteria in DSM-5. A study in the US shows that the changes in the diagnostic criteria for PTSD in the DSM from IV to 5 have had a minimal impact on prevalence[16]. Still, the national PTSD prevalence estimates in the US have been slightly lower (ca 1%), for both lifetime, and past 12-month PTSD when using DSM-5 as compared with DSM-IV[16]. In the same study, the differences between DSM-IV and DSM-5 seem to be due to the exclusion of the "sudden unexpected

For peer review only - http://bmjopen.bmj.com/site/about/guidelines.xhtml

death of a loved one" as criteria for trauma in the DSM-5 and that DSM-5 is more explicit regarding the avoidance critera[16]. The altered trauma criteria and the more explicit avoidance criteria seems to make the greatest difference between DSM-IV and DSM-5 in our study too.

This study has found that the validity of the PTSD diagnoses in the health care registers of the Region of Stockholm is sufficient for epidemiological research. The register can be used for population-based register studies of PTSD for men and women, Swedish born and migrants and person with and without psychosis according to MR and studies generated from its use have the potential to greatly improve understanding of the prevalence and incidence of PTSD and risk factors of the diagnosis.

Author's contributions: A-CH and CD conceived the study. A-CH and CD designed the study and obtained funding from FORTE. A-CH, CD and SW acquired register with the diagnoses and access to the MRs. A-CH and SW prepared the data including the randomised sample. KA and CE validated the diagnoses. EH supervised KA and CE regarding PTSD. A-CH wrote the study protocol. A-CH drafted the data tables. A-CH and SW did the statistical analyses. ACH wrote the manuscript. All authors critically revised the paper for important intellectual content and approved the final version.

The original protocol for the study is provided as a supplementary file.

Funding statement: This work was supported by FORTE (dnr 2016-00870).

Competing interests statement: 'None declared'

Any checklist and flow diagram for the appropriate reporting statement, the STRAND, see supplementary file.

Data sharing statement: Under Swedish law and ethical approval, patient level data cannot be made available.

Patient involvement: No patients were involved in setting the research question or the outcome measures, nor were they involved in developing plans for design or implementation of the study. No patients were asked to advise on interpretation or writing up of results.

Acknowledgments: We are grateful of the heads of the local specialist psychiatric treatment centres that gave permission to use the MRs from their centres in this study.

For peer review only - http://bmjopen.bmj.com/site/about/guidelines.xhtml

References

1. Bisson JI, Cosgrove S, Lewis C, et al. Post-traumatic stress disorder. *BMJ* 2015;351:h6161. doi: 10.1136/bmj.h6161
2. American Psychiatric Association, American Psychiatric Association. DSM-5 Task Force. Diagnostic and statistical manual of mental disorders: DSM-5. 5. ed. Arlington, Va.: American Psychiatric Association 2013.
3. American Psychiatric Association, American Psychiatric Association. Task Force on DSM-IV. Diagnostic and statistical manual of mental disorders: DSM-IV-TR. 4. ed. Washington, DC: American Psychiatric Association 2000.
4. Duckers ML, Alisic E, Brewin CR. A vulnerability paradox in the cross-national prevalence of post-traumatic stress disorder. *Br J Psychiatry* 2016;209(4):300-05. doi: 10.1192/bjp.bp.115.176628
5. Burri A, Maercker A. Differences in prevalence rates of PTSD in various European countries explained by war exposure, other trauma and cultural value orientation. *BMC Res Notes* 2014;7:407. doi: 10.1186/1756-0500-7-407
6. Seow LSE, Ong C, Mahesh MV, et al. A systematic review on comorbid post-traumatic stress disorder in schizophrenia. *Schizophrenia Research* 2016;176(2-3):441-51. doi: 10.1016/j.schres.2016.05.004
7. Brand RM, Rossell SL, Bendall S, et al. Can We Use an Interventionist-Causal Paradigm to Untangle the Relationship between Trauma, PTSD and Psychosis? *Front Psychol* 2017;8:306. doi: 10.3389/fpsyg.2017.00306
8. Hollander AC, Dal H, Lewis G, et al. Refugee migration and risk of schizophrenia and other non-affective psychoses: cohort study of 1.3 million people in Sweden. *BMJ* 2016;352:i1030. doi: 10.1136/bmj.i1030
9. Schouten BC, Meeuwesen L. Cultural differences in medical communication: a review of the literature. *Patient Educ Couns* 2006;64(1-3):21-34. doi: 10.1016/j.pec.2005.11.014
10. Allebeck P. The use of population based registers in psychiatric research. *Acta Psychiatrica Scandinavica* 2009;120(5):386-91. doi: 10.1111/j.1600-0447.2009.01474.x
11. Ludvigsson JF, Andersson E, Ekbom A, et al. External review and validation of the Swedish national inpatient register. *BMC Public Health* 2011;11:450. doi: 1471-2458-11-450 [pii]
12. World Health Organization. International statistical classification of diseases and related health problems [Elektronisk resurs] : ICD-10. 2. ed. Geneva: World Health Organization 2005.
13. Okkels N, Trabjerg B, Arendt M, et al. Traumatic Stress Disorders and Risk of Subsequent Schizophrenia Spectrum Disorder or Bipolar Disorder: A Nationwide Cohort Study. *Schizophr Bull* 2017;43(1):180-86. doi: 10.1093/schbul/sbw082
14. Sheehan DV, Lecrubier Y, Sheehan KH, et al. The Mini-International Neuropsychiatric Interview (M.I.N.I.): the development and validation of a structured diagnostic psychiatric interview for DSM-IV and ICD-10. *J Clin Psychiatry* 1998;59 Suppl 20:22-33;quiz 34-57.
15. Gravely AA, Cutting A, Nugent S, et al. Validity of PTSD diagnoses in VA administrative data: comparison of VA administrative PTSD diagnoses to self-reported PTSD Checklist scores. *J Rehabil Res Dev* 2011;48(1):21-30.
16. Kilpatrick DG, Resnick HS, Milanak ME, et al. National estimates of exposure to traumatic events and PTSD prevalence using DSM-IV and DSM-5 criteria. *J Trauma Stress* 2013;26(5):537-47. doi: 10.1002/jts.21848

For peer review only - http://bmjopen.bmj.com/site/about/guidelines.xhtml

Appendix A: Each specific diagnose criteria of PTSD according to the DSM-IV and DSM-5 and the percent fulfilment of each of these criteria according to the MRs of the 187 MRs

	**DSM-IV**		**DSM-5**	
	Criteria		Criteria	
A	Exposed to a traumatic event	93%	The person was exposed to: death, threatened death, actual or threatened serious injury, or actual or threatened sexual violence	92%
B	The traumatic event is persistently re-experienced	96%	The traumatic event is persistently re-experienced	96%
C	Persistent avoidance of stimuli associated with the trauma and numbing of general responsiveness	88%	Avoidance of trauma-related stimuli after the trauma	84%
D	Persistent symptoms of increased arousal	96%	Negative thoughts or feelings that began or worsened after the trauma	94%
E	Symptoms last for more than 1 month.	98%	Trauma-related arousal and reactivity that began or worsened after the trauma	97%
F	The disturbance causes clinically significant distress or impairment	98%	Symptoms last for more than 1 month.	98%
G		n.a.	Symptoms create distress or functional impairment	97%
H		n.a.	Symptoms are not due to medication, substance use, or other illness.	93%

For peer review only

Section & Topic	No	Item	Reported on page #
**TITLE OR ABSTRACT**			
	**1**	Identification as a study of diagnostic accuracy using at least one measure of accuracy (such as sensitivity, specificity, predictive values, or AUC)	2
**ABSTRACT**			
	**2**	Structured summary of study design, methods, results, and conclusions (for specific guidance, see STARD for Abstracts)	2
**INTRODUCTION**			
	**3**	Scientific and clinical background, including the intended use and clinical role of the index test	3
	**4**	Study objectives and hypotheses	4
**METHODS**			
Study design	**5**	Whether data collection was planned before the index test and reference standard were performed (prospective study) or after (retrospective study)	4
Participants	**6**	Eligibility criteria	4
	**7**	On what basis potentially eligible participants were identified (such as symptoms, results from previous tests, inclusion in registry)	4
	**8**	Where and when potentially eligible participants were identified (setting, location and dates)	4
	**9**	Whether participants formed a consecutive, random or convenience series	4
Test methods	**10a**	Index test, in sufficient detail to allow replication	4
	**10b**	Reference standard, in sufficient detail to allow replication	4
	**11**	Rationale for choosing the reference standard (if alternatives exist)	7
	**12a**	Definition of and rationale for test positivity cut-offs or result categories of the index test, distinguishing pre-specified from exploratory	na
	**12b**	Definition of and rationale for test positivity cut-offs or result categories of the reference standard, distinguishing pre-specified from exploratory	na
	**13a**	Whether clinical information and reference standard results were available to the performers/readers of the index test	na
	**13b**	Whether clinical information and index test results were available to the assessors of the reference standard	na
Analysis	**14**	Methods for estimating or comparing measures of diagnostic accuracy	5
	**15**	How indeterminate index test or reference standard results were handled	na
	**16**	How missing data on the index test and reference standard were handled	na
	**17**	Any analyses of variability in diagnostic accuracy, distinguishing pre-specified from exploratory	5
	**18**	Intended sample size and how it was determined	4
**RESULTS**			
Participants	**19**	Flow of participants, using a diagram	5
	**20**	Baseline demographic and clinical characteristics of participants	5
	**21a**	Distribution of severity of disease in those with the target condition	na
	**21b**	Distribution of alternative diagnoses in those without the target condition	5
	**22**	Time interval and any clinical interventions between index test and reference standard	na
Test results	**23**	Cross tabulation of the index test results (or their distribution) by the results of the reference standard	6
	**24**	Estimates of diagnostic accuracy and their precision (such as 95% confidence intervals)	6
	**25**	Any adverse events from performing the index test or the reference standard	na
**DISCUSSION**			
	**26**	Study limitations, including sources of potential bias, statistical uncertainty, and generalisability	7
	**27**	Implications for practice, including the intended use and clinical role of the index test	7
**OTHER INFORMATION**			
	**28**	Registration number and name of registry	na
	**29**	Where the full study protocol can be accessed	8
	**30**	Sources of funding and other support; role of funders	8

For peer review only

CC BY SA

STARD 2015

AIM

STARD stands for "Standards for Reporting Diagnostic accuracy studies". This list of items was developed to contribute to the completeness and transparency of reporting of diagnostic accuracy studies. Authors can use the list to write informative study reports. Editors and peer-reviewers can use it to evaluate whether the information has been included in manuscripts submitted for publication.

EXPLANATION

A **diagnostic accuracy study** evaluates the ability of one or more medical tests to correctly classify study participants as having a **target condition.** This can be a disease, a disease stage, response or benefit from therapy, or an event or condition in the future. A medical test can be an imaging procedure, a laboratory test, elements from history and physical examination, a combination of these, or any other method for collecting information about the current health status of a patient.

The test whose accuracy is evaluated is called **index test.** A study can evaluate the accuracy of one or more index tests. Evaluating the ability of a medical test to correctly classify patients is typically done by comparing the distribution of the index test results with those of the **reference standard**. The reference standard is the best available method for establishing the presence or absence of the target condition. An accuracy study can rely on one or more reference standards.

If test results are categorized as either positive or negative, the cross tabulation of the index test results against those of the reference standard can be used to estimate the **sensitivity** of the index test (the proportion of participants *with* the target condition who have a positive index test), and its **specificity** (the proportion *without* the target condition who have a negative index test). From this cross tabulation (sometimes referred to as the contingency or "2x2" table), several other accuracy statistics can be estimated, such as the positive and negative **predictive values** of the test. Confidence intervals around estimates of accuracy can then be calculated to quantify the statistical **precision** of the measurements.

If the index test results can take more than two values, categorization of test results as positive or negative requires a **test positivity cut-off**. When multiple such cut-offs can be defined, authors can report a receiver operating characteristic (ROC) curve which graphically represents the combination of sensitivity and specificity for each possible test positivity cut-off. The **area under the ROC curve** informs in a single numerical value about the overall diagnostic accuracy of the index test.

The **intended use** of a medical test can be diagnosis, screening, staging, monitoring, surveillance, prediction or prognosis. The **clinical role** of a test explains its position relative to existing tests in the clinical pathway. A replacement test, for example, replaces an existing test. A triage test is used before an existing test; an add-on test is used after an existing test.

Besides diagnostic accuracy, several other outcomes and statistics may be relevant in the evaluation of medical tests. Medical tests can also be used to classify patients for purposes other than diagnosis, such as staging or prognosis. The STARD list was not explicitly developed for these other outcomes, statistics, and study types, although most STARD items would still apply.

DEVELOPMENT

This STARD list was released in 2015. The 30 items were identified by an international expert group of methodologists, researchers, and editors. The guiding principle in the development of STARD was to select items that, when reported, would help readers to judge the potential for bias in the study, to appraise the applicability of the study findings and the validity of conclusions and recommendations. The list represents an update of the first version, which was published in 2003.

More information can be found on http://www.equator-network.org/reporting-guidelines/stard.

For peer review only

CC BY SA

BMJ Open

A validation study of randomly selected cases of PTSD diagnoses identified in a Swedish regional database compared with medical records - Is the validity sufficient for epidemiological research?

Journal:	*BMJ Open*
Manuscript ID	bmjopen-2019-031964.R1
Article Type:	Original research
Date Submitted by the Author:	19-Nov-2019
Complete List of Authors:	Hollander, Anna-Clara; Karolinska Institutet, Department of Public Health Sciences<br>Askegård, Klara; Psykiatri Södra, Administration, Tideliusgatan 12, plan 6, 118 69 Stockholm<br>Escalante-Bernal, Claudia; Psykiatri Södra, Administration, Tideliusgatan 12, plan 6, 118 69 Stockholm<br>Holmes, Emily; Karolinska Institutet Department of Clinical Neuroscience; Uppsala University, Sweden, Department of Psychology<br>Wicks, Susanne; Karolinska Institutet, Division of Public Health Epidemiology, Department of Public Health Sciences; Centre for Epidemiology and Community Medicine, Stockholm County Council, SE-171 77 Stockholm, Sweden<br>Dalman, Christina; Karolinska Institute, Public Health Sciences; Centre for Epidemiology and Community Medicine, Stockholm County Council, SE-171 77 Stockholm, Sweden
**Primary Subject Heading**:	Mental health
Secondary Subject Heading:	Diagnostics, Epidemiology
Keywords:	EPIDEMIOLOGY, PSYCHIATRY, PUBLIC HEALTH

SCHOLARONE™
Manuscripts

Title page

Title: A validation study of randomly selected cases of PTSD diagnoses identified in a Swedish regional database compared with medical records - Is the validity sufficient for epidemiological research?

Corresponding author: Anna-Clara Hollander, Department of Public Health Sciences, Karolinska Institutet, 171 77 Stockholm, Sweden, anna-clara.hollander@ki.se

Authors and affiliations:

Anna-Clara Hollander[1], Klara Askegård[2], Claudia Escalante[2], Emily A. Holmes[3], Susanne Wicks[1,4], Christina Dalman[1,4]

[1]Epidemiology of Psychiatric Conditions, Substance use and Social environment (EPiCSS), Department of Public Health Sciences, Karolinska Institutet, 171 77 Stockholm

[2]Psykiatri Södra, Administration, Tideliusgatan 12, plan 6, 118 69 Stockholm

[3]Emotional Mental Imagery Lab (EMIL), Department of Clinical Neuroscience, Karolinska Institutet, 171 77 Stockholm and Department of Psychology, Uppsala University, Sweden

[4]Centre for Epidemiology and Community Medicine, Stockholm County Council, SE-171 77 Stockholm, Sweden

Word count: 2536

For peer review only

For peer review only - http://bmjopen.bmj.com/site/about/guidelines.xhtml

Abstract

Objectives: In Sweden, the patients' diagnoses are recorded in administrative registers. The research value of these registers is determined by their diagnostic validity, i.e. if the diagnosis recorded meets the relevant diagnostic criteria. The aim of the study was to assess the validity of PTSD-diagnoses as compared with case notes in medical records (MR) and to test if there was a difference in validity by gender, migration status and those with and without psychotic symptoms. We hypothesized that the validity would be sufficient, using both DSM-IV and DSM-5 but higher according to DSM-IV than DSM-5, and that the validity would be the same for men and women, but different for Swedish-born and migrants, and for those with and without psychotic symptoms.

Design and setting: A validation of the register-diagnoses using MRs from treatment centres within the Region of Stockholm to examine whether patients with a register-diagnosis of PTSD fulfilled DSM criteria of PTSD according to the case notes in their MRs.

Participants: A random sample of 187 patients aged 18-64, who had been diagnosed with PTSD (F43.1 in the ICD-10) were drawn from the Region of Stockholm's MR database 2013 – 2015.

Primary outcome measure: Validity of the PTSD diagnoses according to DSM-IV and DSM-5 as proportions of true positives with 95% confidence interval.

Results: The hypothesised sufficient validity of the PTSD diagnoses was confirmed. Although the point-estimates for DSM-IV were higher than for DSM-5, the hypothesis that there would be significant differences in validity between DSM-IV and DSM-5 was not confirmed. There were no significant validity differences by gender, migration status and for those with and without psychotic symptoms.

Conclusions: This study has found that validity of the PTSD diagnoses in the register of the Region of Stockholm to be sufficient for epidemiological research.

Keywords: post-traumatic stress disorder, diagnostic validity, administrative registers, DSM IV, DSM-5

Strengths and limitations of this study

- Randomly selected sample of cases with a PTSD diagnosis from the comprehensive register of the Stockholm region with an almost complete coverage and mixed urban populations.
- Thorough review of the medical records performed by two medical doctors.
- The two medical doctors reviewing medical records had a high coherence.
- The method of validating diagnosis by reviewing MRs carries a risk of misinterpretation when reading another medical doctor's case notes.

For peer review only - http://bmjopen.bmj.com/site/about/guidelines.xhtml

For peer review only

- The Stockholm population is an urban population, hence there might be specific aspects of PTSD in rural areas that are not taken into account.

For peer review only

For peer review only - http://bmjopen.bmj.com/site/about/guidelines.xhtml

A validation study of randomly selected cases of PTSD diagnoses identified in a Swedish regional database compared with medical records - Is the validity sufficient for epidemiological research?

Introduction

Posttraumatic stress disorder (PTSD) can develop after a person has been exposed to exceptionally threatening or horrifying events which qualify as "psychological trauma"[1]. Symptoms of PTSD, continuing more than a month after the event, include involuntary, and intrusive upsetting memories of the traumatic event, thoughts, feelings, or dreams related to the events, mental or physical distress to trauma-related cues, attempts to avoid trauma-related cues, alterations in how a person thinks and feels, and increased arousal[2 3]. There is a strong case for the cross-cultural validity of PTSD[4], still, estimates of PTSD prevalence differ greatly between countries[4]. In a study of PTSD in European countries, the highest prevalence of PTSD was found in war-torn Croatia, followed by the Netherlands, the UK, France and Germany[5]. The samples included in this comparison were small and survey-based with varying attrition.

Psychotic disorders and PTSD are different in many ways but arguably have some similarities in terms of specific symptoms[6]. Some avoidance behaviours in PTSD resemble safety-seeking behaviours or negative symptoms in psychosis[6]. Hallucinations in psychosis have similarities to the experience of flashbacks and intrusive images and bodily sensations in PTSD[6]. There is an ongoing discussion about the relationship between PTSD and psychosis[7]. Studies of refugees have, so far, often had a focus on PTSD, however, a study from 2016 showed that in particular male refugees also have an increased risk of non-affective psychosis[8]. This study has intensified an already ongoing debate regarding the validity of PTSD diagnoses among male and female migrants, especially with psychosis, as for mental health professionals, different cultural variation in presentation of psychiatric symptoms contributes to risk of being misdiagnosed[9].

In Scandinavia and Finland, visits to psychiatric care are recorded in local and national administrative registers covering the entire population (also known as population-based registers). Such registers have benefited mental health research immensely[10]. Central understanding regarding major mental disorders, for instance, schizophrenia, bipolar disorder, suicide, autism spectrum disorders (ASD) would not exist without the use of population based registers[10]. When using these administrative registers created for generic purposes in research it is crucial to assess the quality of the information they contain, i.e. testing the diagnostic validity[11]. Diagnostic validity refers, in this context, to the accuracy of a diagnose, if the diagnoses recorded meets the relevant diagnostic criteria, measured as proportions of true positives. The diagnose is considered valid if it corresponds to the diagnostic criteria according to either *International Classification of Diseases* (ICD)[12] or *Diagnostic and Statistical Manual of Mental Disorders* (DSM)[2 3 12]. In Sweden diagnoses are registered according to the ICD-10. The share of cases with a valid diagnosis differ for different diagnoses and for different registers but in Sweden the population registers who have been validated have had a validity around 85-95%[11], hence this level can be considered as a sufficient validity. Validation studies have been performed for a range of specific psychiatric diagnoses e.g. schizophrenia, ASD, bipolar disorder etc.[11], in the Swedish registers, but hitherto the diagnosis of PTSD has not been validated. A small validation study of PTSD was performed in Denmark 2015 testing the validity of PTSD[13] defined according to ICD-10[12]. The study used 18 cases of PTSD from the Danish Psychiatric Central Research Register and found a positive predictive value of 83%. The PTSD diagnosis are of special interest since the DSM criteria for

For peer review only - http://bmjopen.bmj.com/site/about/guidelines.xhtml

PTSD have been changed recently. Valid PTSD-diagnoses that can be used in population-based register studies could improve understanding of the prevalence and incidence of PTSD.

The first aim of the current study is to assess the validity of PTSD-diagnoses in a regional register as compared with case notes in medical records (MR) according to the DSM-IV and DSM-5 criterion, in order to determine if the register diagnoses are of sufficient quality for epidemiological research. We hypothesize that the validity of PTSD-diagnoses will be sufficient, using both DSM-IV and DSM-5 but that the validity would be higher according to DSM-IV than the DSM-5, as for the time frame chosen more clinicians would have been familiar with DSM-IV. The second aim is to test if there is a difference in the validity by gender, migration status and among the patients with and without psychotic symptoms. We hypothesize that the validity will be the same for men and women, but different for Swedish-born and migrants and for those with and without psychotic symptoms, with a higher validity for Swedish born and those without psychotic symptoms.

Method

Setting and design:

The design of the validation was to examine whether patients with register-diagnoses of PTSD fulfilled the DSM criteria of PTSD according to the case notes in their MRs. We chose to assess the diagnoses in accordance with the DSM-system since it has become the global standard in psychiatric research and contains specific criteria for each diagnosis. MRs were retrieved from four local specialist psychiatric treatment centres within the Region of Stockholm in Sweden: Psykiatri Södra, Psykiatri Sydväst, Norra Stockholms Psykiatri and Psykiatri Nordväst.

Population and data source:

We selected a random sample of 200 patients from the approximately 2000 eligible patients in the register, aged 18-64, who had been diagnosed with PTSD (F43.1 according to ICD-10[12]) at one of the above-mentioned local centres as a primary or secondary diagnosis according to the Region of Stockholm's health care register 2013 – 2015. The reason for including 200 patients were to have the statistical power to do the sub-group calculations even if the validity was found to be low. The MRs of these patients were retrieved from the Region of Stockholm's electronic MR database *Take Care,* that was introduced within the Region of Stockholm in 2008 and used by the majority of clinics by year 2010. Thirteen MRs were not possible to retrieve due to, for instance, hidden identity of the MR holder. These MRs were deducted from the total number of MRs. The size of the medical records ranged from 1 - 100 pages, sometimes from different psychiatric caregivers.

Validation procedure:

Two medical doctors, in their psychiatric specialty training, revised 90 MRs each and 20 together to cross-validate their judgements (total n=200). They reviewed both symptoms for PTSD, and psychosis. Considering that DSM-IV was used until late 2014, when DSM-5 was published in Swedish, the PTSD diagnostic criteria was scrutinised for both DSM-IV and DSM-5. Twenty patients were cross validated by both the clinicians in order to calculate the degree of coherence between the two doctors, and in conjunction with supervision from a specialist in PTSD. When the MRs included results from a Mini International Neuropsychiatric Interview (M.I.N.I.)[14] concluding that the patient fulfilled the criteria for a PTSD diagnosis, this was considered as the criteria were fulfilled, although each criterion was not commented on specifically. The clinicians also reviewed the MRs for positive

For peer review only - http://bmjopen.bmj.com/site/about/guidelines.xhtml

or negative symptoms of psychosis, whether the patient fulfilled a psychosis diagnosis according to M.I.N.I. or whether the patient had received any diagnosis of psychosis in the MR (henceforth referred to as psychosis according to MR).

Subgroups

The diagnoses were compared by gender according to the MR, migration status (born in Sweden or not) according to the MR and by psychosis according to notes in the MR. The patient was classified as having psychosis according to notes in the MR by the reviewer if the patients either fulfilled the criteria of both hallucinations and delusions or were diagnosed with a psychotic disorder according to M.I.N.I[14] or if the patient was classified as having a suspected psychotic diagnosis.

Statistical methods:

We calculated the degree of coherence between raters, in percent and validity as positive predictive value (ppv) among the register diagnoses with 95% confidence interval (95% CI), ppv is defined as number of patients with a PTSD register diagnosis confirmed in the medical records divided by the total number of patients with a PTSD register diagnosis that we were able to validate against medical records. The differences between subgroups were compared using chi-square tests.

In order for a DSM-criteria to be counted as fulfilled, the criteria needed to be explicitly mentioned in the MR. However, criteria C for PTSD (the avoidance criteria) seemed sometimes not to be mentioned unless it was not fulfilled (i.e. “the patient do not seem to avoid related cues”). An additional test of validity was made, were we counted criteria C to be fulfilled each time it was not specifically described as not to be fulfilled.

Ethics:

The study was approved by the Regional Ethical Review Board in Stockholm (Dnr 2016/1537-32).

Results:

Study population

Due to either protected identities or inaccessible records, MRs could not be validated for 13 persons out of the 200 selected. Since we could not assess the validity of PTSD among these patients, they were excluded from the study. Altogether 187 register diagnoses were validated in the final sample, which included more women than men and more Swedish born than migrants, see table 1. The coherence between the two medical doctors validating the MRs was 80% (95% CI =62-98%) for DSM-IV and 85% (95% CI=69-100%) for DSM-5.

Table 1: Demographic description of the retrieved cases of PTSD from the register

		**N (%)**
**Total number of selected cases**		200 (100%)
Number of accessible records i.e. final sample		187 (94%)
**Demographics of the final sample**		
Gender according to MR	**Men**	68 (36%)
	**Women**	119 (64%)
Migration status according to MR	**Swedish born**	108 (58%)
	**Foreign born**	79 (42%)
Psychosis according to notes in the MR	**Yes**	16 (9%)

For peer review only - http://bmjopen.bmj.com/site/about/guidelines.xhtml

	**No**	171 (91%)

The validity of PTSD according to DSM-IV and DSM 5

Out of 187 patients, 84% (95% CI =79-90%) fulfilled the criteria of PTSD according to DSM-IV and 75% (95% CI =69-82%) of the patients qualified for a PTSD diagnosis according to DSM-5, see table 2. There were 29 (for DSM-IV) and 46 (for DSM-5) false positive cases. Among the false positive cases, there were no transferring errors (i.e. complete mistake with unrelated diagnoses miscoded as PTSD) as all cases had some symptoms relating to PTSD, but still did not fulfil the PTSD criteria.

Table 2: Validity, positive predictive value (ppv), of the PTSD-diagnoses in the register among the 187 accessible MRs and the additional test of validity counting cases with inadequate information on criteria C as true positives

	**DSM-IV**		**DSM-5**	
**According to the 187 MRs**	n	ppv (95% CI)	n	ppv (95% CI)
True positive cases	158	84 (79-90)	141	75 (69-82)
Additional test of validity				
True positive cases	168	90 (86-94)	154	82 (77-88)

The false positive cases were primarily due to two reasons. Either not fulfilling the central criteria of being exposed to a trauma specific event (criteria A) or not describing any signs of avoidance, criteria C. According to the DSM-IV criteria A was fulfilled 93% of the times and 92% the DSM-5. Regarding criteria C, 88% fulfilled the DSM-IV, and 84% the DSM-5 fulfilled criteria C. For more information on the percent of the cases fulfilling each specific PTSD criteria in detail, see Appendix A. In the additional test of validity test criteria C was calculated as fulfilled each time it was not specifically described as not to be fulfilled. When counting validity according to the additional test of validity test the validity of the PTSD-diagnosis was 90% (95% CI =86-94%) for DSM- IV and, 82% (95% CI =77-88%) for DSM- 5. There were no significant differences in terms of validity by gender, migration status and psychosis according to notes in the MR, see table 3.

Table 3: Validity, positive predictive value (ppv), of the PTSD-diagnoses in the register according to accessible MRs by gender, migration status and psychosis according to notes in MR for DSM IV and DSM-5 and p-value for the differences using Chi-square (χ^2) or Fishers exact test.

		**DSM-IV**		**DSM-5**	
		**ppv** (95% CI)	**P-value**	**ppv** (95% CI)	**P-value**
**Gender according to MR**	Men	87 (79-95)	Chi-square Test	78 (68-88)	χ^2-test
	Women	83 (76-90)	0.5163	74 (66-82)	0.5421
**Migration status according to MR**	Swedish born	82 (74-91)	Chi-square Test	72 (62-82)	χ^2-test
	Foreign born	86 (79-93)	0.4745	78 (70-86)	0.3776
**Psychosis according to notes in MR**	Yes	94 (82-100)	Fisher's Exact Test	88 (71-100)	Fisher's Exact Test
	No	84 (78-89)	0.4737	74 (68-81)	0.3648

Discussion

For peer review only - http://bmjopen.bmj.com/site/about/guidelines.xhtml

In this study, the first to assess the validity of PTSD-diagnoses in a Swedish population-based register, the hypothesised sufficient validity of the PTSD-diagnoses was confirmed because the positive predictive value of the diagnoses were between 75-90%. Although the point-estimates for DSM-IV was higher than for DSM-5 the hypothesis that it would be a significant difference in validity between DSM-IV and DSM-5 was not confirmed. There were neither any significant validity differences between men and women, Swedish born and migrants, nor for those with and without psychosis according to notes in the MR.

A strength of the study is that the sample of persons with a PTSD diagnosis was randomly selected from the comprehensive register of the Stockholm country council representing four major psychiatric treatment centres with an almost complete coverage and mixed urban populations, and this limits sampling bias. Another strength is that the clinicians reviewing medical records had a high coherence. A limiting factor with the method of validating diagnosis by reviewing MRs is a risk of misinterpretation when reading the case notes of other medical professionals. The ultimate gold standard of validation is always clinical interviews; however, using MRs is a standard method that as the data is more easily available, more cost effective, and less intrusive for patients.

Criteria C for DSM-IV ("Persistent avoidance of stimuli associated with the trauma and numbing of general responsiveness"), and criteria D for DSM-5 ("Negative thoughts or feelings that began or worsened after the trauma") were considered to be non-stringent by the medical doctors reading the MRs as there is a risk of mistaking these symptoms for a symptom of a depressive state or vice-versa. The Stockholm population is an urban population, hence there might be specific rural aspects of PTSD that are not taken into account. It is also important to note that these are clinical cases; hence, using PTSD in the register only accounts for clinical cases of PTSD and will by definition miss patients who do have PTSD but does not present in the health care system. Another weakness was that the migrant status was defined according to birthplace classified according to the MRs, and not according to a population data base were all places of births are recorded. Most MRs are very detailed in terms of background information however there might be instances were a migration background is not mentioned.

Just like the smaller Danish validation study[13] from 2015 testing the validity of PTSD defined according ICD-10[12] we found a sufficient validity of the PTSD diagnoses. The accuracy we found for DSM-IV (84%) was similar to that of the Danish study (83%) but a little bit lower for DSM-5 (75%), which is perhaps not surprising as it had only just been started to be used in the time frame of our investigation. However, a validation study from the US including 4777 veterans comparing Department of Veterans Affairs administrative data with the self-assessment questioner PTSD Checklist had the same validity as our results as when diagnosed using DSM-5 (75%)[15]. In DSM-5 the American Psychiatric Association updated the diagnostic criteria for PTSD. The key changes between DSM-IV and DSM-5 are that the trauma criterion is different and that feelings such as intense fear, hopelessness, or horror, are removed from DSM-5[16]. Another change is that one criterion was made into two; one avoidance criteria, and one criterion for negative alterations in cognitions and mood. This put more emphasis on avoidance symptoms in the DSM-5 version. Two criterions have also been added, one regarding negative thoughts or feelings and one regarding trauma-related arousal and reactivity, requiring that they began or worsened after the trauma. The validity differences in our study between DSM-IV and DSM-5 were small and not statistically significant and are possibly

For peer review only - http://bmjopen.bmj.com/site/about/guidelines.xhtml

associated with the altered criteria in DSM-5. A study in the US shows that the changes in the diagnostic criteria for PTSD in the DSM from IV to 5 have had a minimal impact on prevalence[16]. Still, the national PTSD prevalence estimates in the US have been slightly lower (ca 1%), for both lifetime, and past 12-month PTSD when using DSM-5 as compared with DSM-IV[16]. In the same study, the differences between DSM-IV and DSM-5 seem to be due to the exclusion of the "sudden unexpected death of a loved one" as criteria for trauma in the DSM-5 and that DSM-5 is more explicit regarding the avoidance critera[16]. The altered trauma criteria and the more explicit avoidance criteria seems to make the greatest difference between DSM-IV and DSM-5 in our study too.

This study has found that the validity of the PTSD diagnoses in the health care registers of the Region of Stockholm is sufficient for epidemiological research. The register can be used for population-based register studies of PTSD for men and women, Swedish born and migrants and person with and without psychosis according to notes in the MR and studies generated from its use have the potential to greatly improve understanding of the prevalence and incidence of PTSD and risk factors of the diagnosis.

Author's contributions: A-CH and CD conceived the study. A-CH and CD designed the study and obtained funding from FORTE. A-CH, CD and SW acquired register with the diagnoses and access to the MRs. A-CH and SW prepared the data including the randomised sample. KA and CE validated the diagnoses. EH supervised KA and CE regarding PTSD. A-CH wrote the study protocol. A-CH drafted the data tables. A-CH and SW did the statistical analyses. ACH wrote the manuscript. All authors critically revised the paper for important intellectual content and approved the final version.

Funding statement: ACH was supported by FORTE (dnr 2016-00870). CD is supported by the Swedish Research Council (grant number 5232010-1052). EAH receives support from the Swedish Research Council (VR) 2017-00957 the Lupina Foundation, and the Oak Foundation (OCAY-18-442).

Competing interests statement: 'None declared'

Data sharing statement: Under Swedish law and ethical approval, patient level data cannot be made available.

Patient involvement: No patients were involved in setting the research question or the outcome measures, nor were they involved in developing plans for design or implementation of the study. No patients were asked to advise on interpretation or writing up of results.

Author Contribution: A-CH and CD conceived the study. A-CH and CD obtained funding. A-CH, CD and SW designed the study. A-CH, CD and SW acquired the data. A-CH and SW prepared the data. KA and CE performed the comparison of the diagnoses and the MR. EAH supervised KA and CE regarding diagnoses accuracy. KA, CE, A-CH and SW conducted the statistical analyses. ACH drafted the data tables. All interpreted statistical analyses. ACH wrote the manuscript. All authors critically revised the paper for important intellectual content and approved the final version.

For peer review only - http://bmjopen.bmj.com/site/about/guidelines.xhtml

Acknowledgments: We are grateful of the heads of the local specialist psychiatric treatment centres that gave permission to use the MRs from their centres in this study. We are grateful of the help of Filip Arnberg, associate professor, lic. clin psychologist, who provided valuable knowledge of the available screening instruments for detecting PTSD.

For peer review only

References

1. Bisson JI, Cosgrove S, Lewis C, et al. Post-traumatic stress disorder. *BMJ* 2015;351:h6161. doi: 10.1136/bmj.h6161
2. American Psychiatric Association, American Psychiatric Association. DSM-5 Task Force. Diagnostic and statistical manual of mental disorders: DSM-5. 5. ed. Arlington, Va.: American Psychiatric Association 2013.
3. American Psychiatric Association, American Psychiatric Association. Task Force on DSM-IV. Diagnostic and statistical manual of mental disorders: DSM-IV-TR. 4. ed. Washington, DC: American Psychiatric Association 2000.
4. Duckers ML, Alisic E, Brewin CR. A vulnerability paradox in the cross-national prevalence of post-traumatic stress disorder. *Br J Psychiatry* 2016;209(4):300-05. doi: 10.1192/bjp.bp.115.176628
5. Burri A, Maercker A. Differences in prevalence rates of PTSD in various European countries explained by war exposure, other trauma and cultural value orientation. *BMC Res Notes* 2014;7:407. doi: 10.1186/1756-0500-7-407
6. Seow LSE, Ong C, Mahesh MV, et al. A systematic review on comorbid post-traumatic stress disorder in schizophrenia. *Schizophrenia Research* 2016;176(2-3):441-51. doi: 10.1016/j.schres.2016.05.004
7. Brand RM, Rossell SL, Bendall S, et al. Can We Use an Interventionist-Causal Paradigm to Untangle the Relationship between Trauma, PTSD and Psychosis? *Front Psychol* 2017;8:306. doi: 10.3389/fpsyg.2017.00306
8. Hollander AC, Dal H, Lewis G, et al. Refugee migration and risk of schizophrenia and other non-affective psychoses: cohort study of 1.3 million people in Sweden. *BMJ* 2016;352:i1030. doi: 10.1136/bmj.i1030
9. Schouten BC, Meeuwesen L. Cultural differences in medical communication: a review of the literature. *Patient Educ Couns* 2006;64(1-3):21-34. doi: 10.1016/j.pec.2005.11.014
10. Allebeck P. The use of population based registers in psychiatric research. *Acta Psychiatrica Scandinavica* 2009;120(5):386-91. doi: 10.1111/j.1600-0447.2009.01474.x
11. Ludvigsson JF, Andersson E, Ekbom A, et al. External review and validation of the Swedish national inpatient register. *BMC Public Health* 2011;11:450. doi: 1471-2458-11-450 [pii]
12. World Health Organization. International statistical classification of diseases and related health problems [Elektronisk resurs] : ICD-10. 2. ed. Geneva: World Health Organization 2005.
13. Svensson E, Lash TL, Resick PA, et al. Validity of reaction to severe stress and adjustment disorder diagnoses in the Danish Psychiatric Central Research Registry. *Clinical Epidemiology* 2015:7 235–242
14. Sheehan DV, Lecrubier Y, Sheehan KH, et al. The Mini-International Neuropsychiatric Interview (M.I.N.I.): the development and validation of a structured diagnostic psychiatric interview for DSM-IV and ICD-10. *J Clin Psychiatry* 1998;59 Suppl 20:22-33;quiz 34-57.
15. Gravely AA, Cutting A, Nugent S, et al. Validity of PTSD diagnoses in VA administrative data: comparison of VA administrative PTSD diagnoses to self-reported PTSD Checklist scores. *J Rehabil Res Dev* 2011;48(1):21-30.
16. Kilpatrick DG, Resnick HS, Milanak ME, et al. National estimates of exposure to traumatic events and PTSD prevalence using DSM-IV and DSM-5 criteria. *J Trauma Stress* 2013;26(5):537-47. doi: 10.1002/jts.21848

For peer review only

For peer review only - http://bmjopen.bmj.com/site/about/guidelines.xhtml

Appendix A: Each specific diagnose criteria of PTSD according to the DSM-IV and DSM-5 and the percent fulfilment of each of these criteria according to the MRs of the 187 MRs

	**DSM-IV**		**DSM-5**	
	Criteria		Criteria	
A	Exposed to a traumatic event	93%	The person was exposed to: death, threatened death, actual or threatened serious injury, or actual or threatened sexual violence	92%
B	The traumatic event is persistently re-experienced	96%	The traumatic event is persistently re-experienced	96%
C	Persistent avoidance of stimuli associated with the trauma and numbing of general responsiveness	88%	Avoidance of trauma-related stimuli after the trauma	84%
D	Persistent symptoms of increased arousal	96%	Negative thoughts or feelings that began or worsened after the trauma	94%
E	Symptoms last for more than 1 month.	98%	Trauma-related arousal and reactivity that began or worsened after the trauma	97%
F	The disturbance causes clinically significant distress or impairment	98%	Symptoms last for more than 1 month.	98%
G		n.a.	Symptoms create distress or functional impairment	97%
H		n.a.	Symptoms are not due to medication, substance use, or other illness.	93%

For peer review only

Section & Topic	No	Item	Reported on page #
**TITLE OR ABSTRACT**			
	**1**	Identification as a study of diagnostic accuracy using at least one measure of accuracy (such as sensitivity, specificity, predictive values, or AUC)	2
**ABSTRACT**			
	**2**	Structured summary of study design, methods, results, and conclusions (for specific guidance, see STARD for Abstracts)	2
**INTRODUCTION**			
	**3**	Scientific and clinical background, including the intended use and clinical role of the index test	3
	**4**	Study objectives and hypotheses	4
**METHODS**			
Study design	**5**	Whether data collection was planned before the index test and reference standard were performed (prospective study) or after (retrospective study)	4
Participants	**6**	Eligibility criteria	4
	**7**	On what basis potentially eligible participants were identified (such as symptoms, results from previous tests, inclusion in registry)	4
	**8**	Where and when potentially eligible participants were identified (setting, location and dates)	4
	**9**	Whether participants formed a consecutive, random or convenience series	4
Test methods	**10a**	Index test, in sufficient detail to allow replication	4
	**10b**	Reference standard, in sufficient detail to allow replication	4
	**11**	Rationale for choosing the reference standard (if alternatives exist)	7
	**12a**	Definition of and rationale for test positivity cut-offs or result categories of the index test, distinguishing pre-specified from exploratory	na
	**12b**	Definition of and rationale for test positivity cut-offs or result categories of the reference standard, distinguishing pre-specified from exploratory	na
	**13a**	Whether clinical information and reference standard results were available to the performers/readers of the index test	na
	**13b**	Whether clinical information and index test results were available to the assessors of the reference standard	na
Analysis	**14**	Methods for estimating or comparing measures of diagnostic accuracy	5
	**15**	How indeterminate index test or reference standard results were handled	na
	**16**	How missing data on the index test and reference standard were handled	na
	**17**	Any analyses of variability in diagnostic accuracy, distinguishing pre-specified from exploratory	5
	**18**	Intended sample size and how it was determined	4
**RESULTS**			
Participants	**19**	Flow of participants, using a diagram	5
	**20**	Baseline demographic and clinical characteristics of participants	5
	**21a**	Distribution of severity of disease in those with the target condition	na
	**21b**	Distribution of alternative diagnoses in those without the target condition	5
	**22**	Time interval and any clinical interventions between index test and reference standard	na
Test results	**23**	Cross tabulation of the index test results (or their distribution) by the results of the reference standard	6
	**24**	Estimates of diagnostic accuracy and their precision (such as 95% confidence intervals)	6
	**25**	Any adverse events from performing the index test or the reference standard	na
**DISCUSSION**			
	**26**	Study limitations, including sources of potential bias, statistical uncertainty, and generalisability	7
	**27**	Implications for practice, including the intended use and clinical role of the index test	7
**OTHER INFORMATION**			
	**28**	Registration number and name of registry	na
	**29**	Where the full study protocol can be accessed	8
	**30**	Sources of funding and other support; role of funders	8

For peer review only - http://bmjopen.bmj.com/site/about/guidelines.xhtml

cc BY SA

STARD 2015

AIM

STARD stands for "Standards for Reporting Diagnostic accuracy studies". This list of items was developed to contribute to the completeness and transparency of reporting of diagnostic accuracy studies. Authors can use the list to write informative study reports. Editors and peer-reviewers can use it to evaluate whether the information has been included in manuscripts submitted for publication.

EXPLANATION

A **diagnostic accuracy study** evaluates the ability of one or more medical tests to correctly classify study participants as having a **target condition.** This can be a disease, a disease stage, response or benefit from therapy, or an event or condition in the future. A medical test can be an imaging procedure, a laboratory test, elements from history and physical examination, a combination of these, or any other method for collecting information about the current health status of a patient.

The test whose accuracy is evaluated is called **index test.** A study can evaluate the accuracy of one or more index tests. Evaluating the ability of a medical test to correctly classify patients is typically done by comparing the distribution of the index test results with those of the **reference standard**. The reference standard is the best available method for establishing the presence or absence of the target condition. An accuracy study can rely on one or more reference standards.

If test results are categorized as either positive or negative, the cross tabulation of the index test results against those of the reference standard can be used to estimate the **sensitivity** of the index test (the proportion of participants *with* the target condition who have a positive index test), and its **specificity** (the proportion *without* the target condition who have a negative index test). From this cross tabulation (sometimes referred to as the contingency or "2x2" table), several other accuracy statistics can be estimated, such as the positive and negative **predictive values** of the test. Confidence intervals around estimates of accuracy can then be calculated to quantify the statistical **precision** of the measurements.

If the index test results can take more than two values, categorization of test results as positive or negative requires a **test positivity cut-off**. When multiple such cut-offs can be defined, authors can report a receiver operating characteristic (ROC) curve which graphically represents the combination of sensitivity and specificity for each possible test positivity cut-off. The **area under the ROC curve** informs in a single numerical value about the overall diagnostic accuracy of the index test.

The **intended use** of a medical test can be diagnosis, screening, staging, monitoring, surveillance, prediction or prognosis. The **clinical role** of a test explains its position relative to existing tests in the clinical pathway. A replacement test, for example, replaces an existing test. A triage test is used before an existing test; an add-on test is used after an existing test.

Besides diagnostic accuracy, several other outcomes and statistics may be relevant in the evaluation of medical tests. Medical tests can also be used to classify patients for purposes other than diagnosis, such as staging or prognosis. The STARD list was not explicitly developed for these other outcomes, statistics, and study types, although most STARD items would still apply.

DEVELOPMENT

This STARD list was released in 2015. The 30 items were identified by an international expert group of methodologists, researchers, and editors. The guiding principle in the development of STARD was to select items that, when reported, would help readers to judge the potential for bias in the study, to appraise the applicability of the study findings and the validity of conclusions and recommendations. The list represents an update of the first version, which was published in 2003.

More information can be found on http://www.equator-network.org/reporting-guidelines/stard.

For peer review only - http://bmjopen.bmj.com/site/about/guidelines.xhtml
