# Supplementary material for: Validation study of randomly selected cases of PTSD diagnoses identified in a Swedish regional database compared with medical records: is the validity sufficient for epidemiological research?
Source: BMJ Open. 2019 Dec 23;9(12):e031964. doi: 10.1136/bmjopen-2019-031964 (PMC7008445; doi:10.1136/bmjopen-2019-031964)
Supplement: Reviewer comments [file bmjopen-2019-031964.reviewer_comments.pdf]

PEER REVIEW HISTORY

BMJ Open publishes all reviews undertaken for accepted manuscripts. Reviewers are asked to complete a checklist review form (**http://bmjopen.bmj.com/site/about/resources/checklist.pdf**) and are provided with free text boxes to elaborate on their assessment. These free text comments are reproduced below.

ARTICLE DETAILS

**TITLE (PROVISIONAL)**	A validation study of randomly selected cases of PTSD diagnoses identified in a Swedish regional database compared with medical records - Is the validity sufficient for epidemiological research?
**AUTHORS**	Hollander, Anna-Clara; Askegård, Klara; Escalante-Bernal, Claudia; Holmes, Emily; Wicks, Susanne; Dalman, Christina

VERSION 1 - REVIEW

**REVIEWER**	Jaimie Gradus<br>Boston University, USA
**REVIEW RETURNED**	18-Sep-2019

**GENERAL COMMENTS**	This article reports the results of a small validation study of posttraumatic stress disorder diagnoses in a Swedish regional health database. While in general I find enormous value in validation studies of registry-based data, I believe the limitations of this study hinder its contribution to the literature. These are outlined below.<br><br>1) It is unclear to me why data that is coded according to ICD-10 would be validated against diagnostic criteria from another diagnostic classification system (in this case DSM). The symptoms of PTSD are not completely identical across ICD and DSM, so why would we expect ICD-based diagnoses to be valid according to DSM criteria? Further, the symptoms of PTSD changed between DSM-IV and DSM-V, so it is also unclear to me a) why we would expect diagnoses made using one set of symptoms to be valid according to the other and b) how diagnoses made during the DSM-IV time period could possibly be validated according to DSM-V because new symptoms were added to the DSM-V diagnosis that did not exist in the DSM-IV version.<br>2) Why were standard validity measures (e.g., positive predictive value or sensitivity and specificity) not calculated?<br>3) There is another larger validation study of PTSD (and other stress) diagnoses in the Danish national registries that the authors may wish to review:<br>https://www.dovepress.com/validity-of-reaction-to-severe-stress-and-adjustment-disorder-diagnose-peer-reviewed-fulltext-article-CLEP<br>4) Given the focus on validity of diagnoses across different subgroups there should be more information in the introduction about why this is important and why we might expect validity to vary in these groups.

	5) I do not believe “feasible” is a term typically used to describe validity in the epidemiologic literature.<br>6) Was there a particular reason a sample size of 200 was chosen?

VERSION 1 – AUTHOR RESPONSE

Reviewer's Comments to Author:

1) It is unclear to me why data that is coded according to ICD-10 would be validated against diagnostic criteria from another diagnostic classification system (in this case DSM). The symptoms of PTSD are not completely identical across ICD and DSM, so why would we expect ICD-based diagnoses to be valid according to DSM criteria? Further, the symptoms of PTSD changed between DSM-IV and DSM-V, so it is also unclear to me a) why we would expect diagnoses made using one set of symptoms to be valid according to the other and b) how diagnoses made during the DSM-IV time period could possibly be validated according to DSM-V because new symptoms were added to the DSM-V diagnosis that did not exist in the DSM-IV version.

Thank you for giving us the opportunity to clarify this. Swedish psychiatrists use DSM and ICD interchangeably but code diagnoses in medical records using ICD 10 codes. Validity articles of the Swedish registers have traditionally used the DSM criteria (Ludvigsson et al, 2011) as neither the Swedish nor English versions of the ICD-10 contain operational diagnostic criteria, but, instead, they contain narrative descriptions of the disorders, justifying the validation against DSM criteria. We have written ”We chose to assess the diagnoses in accordance with the DSM-system since it has become the global standard in psychiatric research and contains specific criteria for each diagnosis.” The reason for validating against both DSMIV and DSM-5 is that the cases were obtain during the process of implementing DSM-5.

Ludvigsson JF, Andersson E, Ekbom A, et al. External review and validation of the Swedish national inpatient register. BMC Public Health 2011;11:450. doi: 1471-2458-11-450

2) Why were standard validity measures (e.g., positive predictive value or sensitivity and specificity) not calculated?

Using positive predictive value is great advice and we have now changed the validity measure to positive predictive value (ppv) throughout. At page 4 under Statistical methods the wording is now: “We calculated the degree of coherence between raters, in percent and validity as positive predictive value (ppv) among the register diagnoses with 95% confidence interval (95% CI), ppv is defined as number of patients with a PTSD register diagnosis confirmed in the medical records divided by the total number of patients with a PTSD register diagnosis that we were able to validate against medical records.” Table 2 and 3 are now displaying the results in ppvs. However, this did not alter the results or conclusions.

Table 2: Validity, positive predictive value (ppv), of the PTSD-diagnoses in the register among the 187 accessible MRs and the additional test of validity counting cases with inadequate information on criteria C as true positives

	DSM-IV		DSM-5	
According to the 187 MRs	n	ppv (95% CI)	n	ppv (95% CI)

True positive cases	158	84 (79-90)	141	75 (69-82)
Additional test of validity				
True positive cases	168	90 (86-94)	154	82 (77-88)

Table 3: Validity, positive predictive value (ppv), of the PTSD-diagnoses in the register according to accessible MRs by gender, migration status and psychosis according to notes in MR for DSM IV and DSM-5 and p-value for the differences using Chi-square (χ^2) or Fishers exact test.

		DSM-IV		DSM-5	
		ppv (95% CI)	P-value	ppv (95% CI)	P-value
Gender according to MR	Men	87 (79-95)	Chi-square Test	78 (68-88)	χ^2-test
	Women	83 (76-90)	0.5163	74 (66-82)	0.5421
Migration status according to MR	Swedish born	82 (74-91)	Chi-square Test	72 (62-82)	χ^2-test
	Foreign born	86 (79-93)	0.4745	78 (70-86)	0.3776
Psychosis according to notes in MR	Yes	94 (82-100)	Fisher's Exact Test	88 (71-100)	Fisher's Exact Test
	No	84 (78-89)	0.4737	74 (68-81)	0.3648

3) There is another larger validation study of PTSD (and other stress) diagnoses in the Danish national registries that the authors may wish to review: https://www.dovepress.com/validity-of-reaction-to-severe-stress-and-adjustment-disorder-diagnose-peer-reviewed-fulltext-article-CLEP

Thank you for giving us the opportunity to quote this excellent paper. We are quoting it in the end of page 3: "A small validation study of PTSD was performed in Denmark 2015 testing the validity of PTSD[13] defined according to ICD-10[12]. The study used 18 cases of PTSD from the Danish Psychiatric Central Research Register and found a positive predictive value of 83%."

4) Given the focus on validity of diagnoses across different subgroups there should be more information in the introduction about why this is important and why we might expect validity to vary in these groups.

We are now highlighting this clearer wording it as follows: "Studies of refugees have, so far, often had a focus on PTSD, however, a study from 2016 showed that in particular male refugees also have an increased risk of non-affective psychosis[8]. This study has intensified an already ongoing debate regarding the validity of PTSD diagnoses among male and female migrants, especially with psychosis, as for mental health professionals, different cultural variation in presentation of psychiatric symptoms contributes to risk of being misdiagnosed[9]."

5) I do not believe "feasible" is a term typically used to describe validity in the epidemiologic literature.

We have now altered the wording to "sufficient".

6) Was there a particular reason a sample size of 200 was chosen?

Thank you for giving us the opportunity to clarify this. It was chosen in order to have the statistical power to do the sub-group calculations even if the validity was low and we now described this at page four with the wording “The reason for including 200 patients were to have the statistical power to do the sub-group calculations even if the validity was found to be low.”.
